# Ammonium to total nitrogen ratio affects the purslane (*Portulaca oleracea* L.) growth, nutritional, and antioxidant status

**DOI:** 10.1016/j.heliyon.2023.e21644

**Published:** 2023-11-02

**Authors:** Antonios Chrysargyris, Efraimia Hajisolomou, Panayiota Xylia, Nikolaos Tzortzakis

**Affiliations:** Department of Agricultural Sciences, Biotechnology and Food Science, Cyprus University of Technology, 3603, Limassol, Cyprus

**Keywords:** Ammonium to total nitrogen ratio, Antioxidants, Mineral fertilizer, Purslane, Soilless culture

## Abstract

Purslane (*Portulaca oleracea* L.) is a widespread weed, which is greatly appreciated for its high nutritional value. The present work evaluated the effect of different ammonium/total nitrogen ratios (NH_4_/Total N: Nr 0.01–0.15) on growth, physiological and biochemical parameters, and nutrient accumulation in different plant parts of hydroponically grown purslane, under two growing seasons, spring and autumn. Young seedlings of purslane were transferred to a Nutrient Film Technique (NFT) system and they were exposed to different Nr levels. The pH and the electrical conductivity of the nutrient solution were kept constant at 5.8 and 2.3 mS cm^−1^, respectively. After the end of the cultivation periods (19 days for spring and 22 days for autumn), a series of assessments (growth parameters, mineral content in different plant organs, antioxidant status of the plant, etc.) were done. Plant height, leaf number, root fresh weight and plant biomass revealed decreased trends at the higher NH_4_/total N ratios, especially during the autumn growing season. Total phenols, flavonoids and antioxidant capacity appeared increased at Nr ≤ 0.10 during both seasons (autumn and spring), revealing higher nitrogen accumulation rates and increased water and nutrient use efficiency. Purslane plants grown in Nr 0.05–0.10 revealed a less intense oxidative stress, with decreased lipid peroxidation levels that was the result of the activation of both enzymatic (superoxide dismutase, catalase and peroxidase) and non-enzymatic (ascorbic acid) antioxidant capacity of the plant. Increased Nr resulted in the accumulation of potassium, while calcium and magnesium levels in leaves were decreased. Additionally, the greater water use efficiency was measured for plants grown under Nr 0.01–0.05. Therefore, the recommended ammonium/total nitrogen ratio for purslane production of increased yield, improved nutritional value and efficient use of water and nitrogen sources is to employ Nr of 0.05, while additional care should be addressed during autumn periods as plants are subjected to greater impacts of the Nr ratio.

## 1 Introduction

Interactions between fertilizer and water management are at the forefront of crop response to nutrients research. Appropriate irrigation and nitrogen fertilization management are critical for crop development, growth and production. Taking into consideration their effect on any crop, improving irrigation regime and nitrogen application practices is of great importance for sustainable agricultural management [1]. As the agricultural sector consumes considerable amount of freshwater, the scarcity of sufficient irrigation water sources is becoming more than acute. On top of that, the high irrigation costs, the increased fertilizer expenses, and a slew of environmental concerns such as environmental pollution from excessive N use, or overuse of other chemical fertilizers in agricultural production systems, present significant concerns [2]. To that end, hydroponics appears to be the ideal cultivation system not only for crop production without the use of soil, but also for introducing unexplored plant species into intensive cultivation regimes, allowing accurate determination of nutrient concentrations and actual needs for each crop [3]. Additionally, the use of a closed hydroponic system offers significant advantages in terms of reducing chemical leaching, conserving water resources, and promoting sustainable agricultural practices, through nutrients and water recirculation. These features make closed hydroponic system an attractive option for efficient and environmentally friendly food production [4].

Modern production practices involve optimization of nitrogen, in order to optimize crop production and minimize the risk of N leaching, as it is a dominant nutrient for the growth and productivity of any crop [1]. In plant organs, nitrogen is the fourth most abundant element after carbon, oxygen, and hydrogen. It is a structural component of amino acids, which are the building blocks of proteins, enzymes, and numerous hormones; it is required in large quantities for the construction and maintenance of plant cells, as total plant protein contains on average about 12 % N by weight. Inorganic nitrogen is the only nutrient taken up by plants in both an anionic and cationic form, as ammonium (NH_4_^+^) and nitrate (NO_3_^−^) ions, and therefore the NH_4_^+^/N uptake ratio has a strong impact on the total nutrient anion to cation uptake [5]. By changing the ammonium/nitrate ratio in the nutrient solution (NS) supplied to the plants, even if the total N content remains constant, the total cation to anion absorption ratio may be considerably altered. That change will affect the pH of the NS and will have an immediate impact on the NS's ability to make macro- and micronutrients available to the plant [6]. When nitrogen fertilizer is used in excess, it can significantly decrease crop yield and nitrogen fertilizer usage efficiency, while at the same time polluting the environment. This imbalanced plant growth pattern will be evident in both the roots and the stems of the plants [7]. The optimal dose of nitrogen fertilizer could greatly raise crop production by boosting nutrient uptake, net assimilation rate, photosynthesis, and dry matter accumulation [8].

Furthermore, overloading the NS with ammonium ion is destructive, as it may increase the intracellular concentration of ammonia, which is highly toxic for the plant cells; many studies have indicated that using ammonium as the sole or dominant N source results in poor growth and restricted yield [5]. On top of that, excessive ammonium in plant tissue disrupts the equilibrium of cations because it forces them to compete each other, dissipating the proton gradient that is necessary for a series of metabolic processes, including photosynthesis, respiration, and transpiration [9]. For example, ammonium ion shares the same ion channels as potassium, and the competitive interactions between them for uptake by the roots, is well evidenced. The nature of these interactions though is complex and depends on the applied concentrations of nitrogen and potassium [6]. According to reports for soilless cultivation, it is recommended that the NH_4_–N levels should not surpass the 25 % of the total nitrogen input. However, this can be revised according to the plant species and/or the applied soilless culture system (close and open hydroponic system) [10]. The addition of ammonium to the NS also prevents the build-up of nitrate inside the plant tissue; increased nitrates content poses a serious threat to human health and causes environmental disruption. Research on the effects of ammonium to nitrate ratio, and consequently ammonium to total nitrogen (Nr = NH_4_^+^/total N), is attracting research interest on leafy vegetables and more lately on medicinal plants [[10], [11], [12], [13]].

Plant species react to NH_4_^+^ in diverse ways, while external conditions as temperature, light level, pH, and the concentration of nutrients in the growing medium can influence these reactions [14]. The nitrogen form that a plant consumes may have an impact on its morphology and chemical uptake; NH_4_^+^ causes lower levels of potassium (K), calcium (Ca), and magnesium (Mg), and increased rates of phosphorus (P), sulphur (S), and organic N [15]. A series of studies have proved that the nitrogen form—rather than the total nitrogen concentration—is more critical, having a considerable impact on overall yield, quality and marketability of fresh produce [16,17]. When both nitrogen forms are available, plants may absorb preferentially one of them, depending on the plant species. Regardless of the fact that NO_3_^−^ uptake and assimilation have a significantly high energetic cost, the majority of plants species favour NO_3_^−^ to NH_4_^+^ [18]. This is because plants may be harmed when are grown in NS with increased levels of NH_4_^+^ (>0.5 mM), which potentially results in reduced biomass synthesis (particularly at low pH levels) [18,19]. On the other hand, an important parameter is the level of nitrate present in the edible plant parts, which poses risks to human health, when consumed; understanding and managing this ratio appropriately can contribute to healthier plants, improved crop yields, and sustainable agricultural practices.

There are many recorded cases of indigenous wild plant species that have been employed by the natives in many Mediterranean countries for ethnomedical or nutritional uses [20]. These plants have been successfully introduced to human diet, due to their vital content in minerals, and because they contain high levels of bioactive substances (such as flavonoids, tannins, vitamins, alkaloids, and phenolic acids) that are major constituent of a healthy diet and potentially maintain or improve human physical condition [21,22]. Even though the majority of these species are traditionally hand-harvested from native populations by the locals, the growing market for such food products has formed a particular niche for the commercialization of indigenous species in order to meet consumer's demands for year-round product offerings and to decrease the likelihood of genetic erosion due to irrational gathering [20]. Therefore, reports for domestication and prospective commercial cultivation of such plants as well as studies about native species' cultivation techniques and how those techniques may affect their biochemical composition and content of bioactive compounds have lately been published [23,24].

*Portulaca oleracea* L., commonly known as purslane, is a wild herb that is popular in the Mediterranean and Asian diets for its crisp, fresh flavour, and has long been a part of both culinary and folk medicinal systems. Purslane's high nutritive and pharmacological properties have been associated with the name “the future superfood” [[25], [26], [27]]. Numerous biological activities are associated to the dietary intake of purslane, including bactericidal, antidiabetic, diuretic, anti-inflammatory, antiseptic, hepatoprotective, neuroprotective, antihyperlipidemic, anti-arthritic and anticancer [26,28,29]. Purslane performed pretty well under several abiotic stresses conditions, including salinity, drought, high temperature levels among others, becoming a potential candidate to perform well under unfavorable conditions [30,31]. Therefore, despite the well reported properties of the species, there is less information on the performance of purslane under an intensive cultivation scheme in hydroponics and the effect of provided nitrogen source. Based on the fact that the species is a leafy vegetable with promising economic interest, the objective of the current study was to investigate how the nitrogen form (ammonium, nitrate) is affecting purslane's performance in terms of plant growth, nutrient content, antioxidant activity, and water and nutrient usage efficiency, under a complete, two-season study.

## 2 Material and methods

### 2.1 Experimental set up and plant material

The experiment took place at the hydroponic infrastructures at the experimental farm of the Cyprus University of Technology, Limassol, Cyprus. A multi-span plastic greenhouse, with a North-South orientation was used, equipped with an automated climatic control system. The greenhouse cover material was transparent polyethylene sheets (anti-drip, resistance to UV radiation, 88 % light transmission). The hydroponic system adapted was the Nutrient Film Technique (NFT), with 12 individual NFT channels (twins white plastic channels, each of 4 m long, 8 cm wide, 6 cm deep) that were aligned with 12 catchments (60 L) to set up 12 independent hydroponic units. Each unit consisted of a twin channel, and it was supported with an individual water replenishment tank (60 L). The nutrient solution (NS) absorbed by the plants was replenished through automatic refill of water from the replenishment tank and the daily adjustment on the NS by using concentrate stock nutrient solution, as described below. Each hydroponic unit accompanied 10 plants, giving a final plant density of 25 plants m^−2^.

The experimental trials were conducted in two distinguished seasons in the NFT system described above, on purslane (*Portulaca oleracea* L.). The first trial took place during autumn (October–November) and the second trial took place during spring (March–April). The effect of four different Nr ratios (ammonium to total nitrogen ratio: Nr) was examined (Nr ratios tested: 0.01, 0.05, 0.10, and 0.15), while the levels of N, K and P were kept constant at 200 mg L^−1^, 350 mg L^−1^ and 70 mg L^−1^, respectively, based on preliminary studies and previous reports [14]. The climatic conditions during the trials were recorded and the air temperature was fluctuated among 19.01 and 31.3 °C during autumn and 18.7 and 33.3 °C during spring.

Commercial purslane seeds were purchased from Greece and were sown in peat based growing media that included 15 % v/v of perlite (to increase media porosity and appropriate drainage), into black multi-plastic trays and were kept under nursery conditions. Young seedlings at the stage of the first true leaf were transplanted into netted pots filled with perlite and were then fitted in the NFT channels spots. Plants were grown for one week under complete NS to allow recovery from the transplant stress. The nutrient concentrations in the standard nutrient solution were as follows: NO_3_^−^-N = 13.57, K^+^ = 8.95, ΡΟ_4_^3-^-P = 2.26, Ca^2+^ = 3.50, Mg^2+^ = 2.88, SO_4_^2-^-S = 1.56 and Na^+^ = 1.93 mmol L^−1^; and Β = 18.52, Fe = 71.56, Μn = 18.21, Cu = 4.72, Zn = 1.53, and Μο = 0.52 μmol L^−1^. Following this first week, plants were subjected to the modified NS with the four Nr ratios for 19 and 22 days, for autumn and spring growing season, respectively (Table S1). Therefore, the tested Nr of 0.01, 0.05, 0.10 and 0.15 ratio were produced by differentiating the NH_4_^+^-N, NO_3_^−^-N and SO_4_^2-^-S levels while keeping constant the total N available to the plants at 200 mg L^−1^ of N. Three independent hydroponic units of the NFT system were used for each ratio, resulting in 3 repetition plots per treatment. The NS was checked every day for the pH and electric conductivity (EC) levels, and was adjusted accordingly, by using H_2_SO_4_ (5 % v/v) for pH maintenance (due to the alkaline water used for the NS preparation), and by adding relevant modified stock solutions –NS, for the EC balance. The target pH and EC of the nutrient solution were 5.8 and 2.3 mS cm^−1^ respectively. Commercial fertilizers used for the preparation of the different nutrient solutions, while all chemical reagents used in the present study were purchased from Sigma Aldrich (Germany), unless it is mentioned differently.

### 2.2 Plant growth measurements

In each experiment, 120 purslane plants were used. After the 19 and 22 days of plant cultivated under the four ammonium/total N ratios (for autumn and spring respectively), six individual plants -from different replication plots from each treatment were considered for the detailed analysis of plant growth features. Plant height, leaf number, leaf, stem, root and total plant fresh and dry weight were determined.

### 2.3 Plant physiology and photosynthetic related parameters

Purslane leaf stomatal conductance was measured by using a Delta-T AP4 dynamic porometer (Delta-T Devices-Cambridge, UK) and expressed as mmol H_2_O m^−2^ s ^−1^. To assess leaf photochemistry, relative chlorophyll content (optical chlorophyll meter SPAD-502, Minolta, Osaka, Japan) and leaf chlorophyll fluorescence (Fv/Fm) were measured with the OptiSci OS-30p Chlorophyll Fluorometer (Opti-Sciences). Moreover, leaf photosynthetic pigments as chlorophyll *a* (Chl a), chlorophyll *b* (Chl b), and total chlorophyll (t-Chl) were analysed. Leaves samples (six replications/treatment, each replication was a pool of leaf tissue from two plants) were incubated in heat bath at 65 °C for 30 min, in the dark, with 10 mL dimethyl sulfoxide (DMSO) for chlorophyll extraction. The content of leaf chlorophyll *a*, chlorophyll *b*, and total chlorophyll was then calculated, using the corresponding equations [32].

To determine electrolyte leakage, leaf tissue was cut into small pieces, in a uniform size, using a round leaf cutter, with a diameter of 10 mm, and placed into a test tube with 20 mL of deionized water. Samples were then incubated at 32 °C for 2 h and then autoclaved at 121 °C for 20 min, to determine the initial electrolyte conductivity (EC_1_) and final EC_2_, respectively, as described by Dionisio-Sese and Tobita [33]. The following formula was used to calculate the electrolyte leakage (EL): EL (%) = $\frac{EC1}{EC2}$ × 100.

### 2.4 Plant organs mineral content and nutrient solution ion concentration analysis

At the completion of the experiment, mineral content in leaves, stems and roots was determined in four replications per treatment (three different plants were pooled for each replication). Samples were dried to constant weight (at 65 °C for 4–5 d) and milled at < 0.42 mm. Sub samples (∼0.45 g) were ashed in porcelain cups at 450 °C for 6 h in an ash furnace (Carbolite, AAF 1100, GERO, Germany). Then, the ash was acid digested with 10 mL hydrochloric acid (2 N HCl). Mineral assessment for potassium, sodium, and phosphorous was performed according to Chrysargyris et al. [34] while magnesium and calcium by an atomic absorption spectrophotometer (PG Instruments AA500FG, Leicestershire, UK). Nitrogen was determined by the Kjeldahl (BUCHI, Digest automat K-439 and Distillation Kjeldahl K-360) method. Data were expressed in g kg^−1^ of dry weight.

The N accumulation rate (AR), N bioaccumulation coefficient (BAC), N translocation factor (TF) and N tolerance index (TI) of purslane were calculated by using the equations described by Benimeli et al. [35], Amin et al. [36] and Azooz et al. [37], as follows.

The N accumulation rate was calculated as the sum up of N content in the tissue of each plant organ x plant DW, divided by the number of days under N levels by the total plant DW [35].

Nitrogen accumulation rate g per kg DW per day

(1) $$=\frac{\left(\left[N\right]\;\text{leave }x\;\text{DW}\;\text{leave}\right)+\left(\left[N\right]\;\text{stem }x\;\text{DW}\;\text{stem}\right)+\left(\left[N\right]\;\text{root }x\;\text{DW}\;\text{root}\right)}{\text{Days }x\;\left(\text{DW}\;\text{leave}+\text{DW}\;\text{stem}+\text{DW}\;\text{root}\right)}$$

The N bioaccumulation coefficient was calculated as the ratio of N content in the tissue of each plant organ of that of N concentration in the nutrient solution, according to Amin et al. [36]:

(2) $$N\;\text{bioaccumulation}\;\text{coefficient}=\frac{N\;\text{content}\;\text{in}\;\text{plant}\;\text{tissue}\;\left(g\;\text{per}\;\text{kg}\;\text{DW}\right)}{N\;\text{concentration}\;\text{in}\;\text{nutrient}\;\text{solution}\;\left(g\;\text{per }L\right)}$$

The translocation factor was calculated as the ratio of N content in the tissue of each plant organ (leaves, stems) to that of N content in plant roots according to Amin et al. [36]:

(3) $$\text{Translocation}\;\text{factor}=\frac{N\;\text{content}\;\text{in}\;\text{plant}\;\text{tissue}\;\left(g\;\text{per}\;\text{kg}\;\text{DW}\right)}{N\;\text{content}\;\text{in}\;\text{plant}\;\text{root}\;\left(g\;\text{per}\;\text{kg}\;\text{DW}\right)}$$

Nitrogen tolerance index was calculated as the quotient of the plant growth-related factor (i.e. total biomass, plant height, leaf number) of treated plants grown under increased Nr and lower Nr conditions, according to the equations described by Benimeli et al. [35] and Azooz et al. [37], with the following modifications:

(4) $$\text{Tolerance}\;\text{index}\;\text{TI }\left(\%\right)=\frac{\text{Growth}-\text{related}\;\text{factor}\;\text{of}\;\text{high}\;\text{Nr}-\text{treated}\;\text{plants }x\;100}{\text{Growth}-\text{related}\;\text{factor}\;\text{of}\;\text{low}\;\text{Nr}-\text{treated}\;\text{plants}}$$

The total amount of water used by the plants during the cropping period was recorded at each hydroponic unit, and water uptake (mL plant^−1^ day^−1^) was calculated. The total amount of N used by the plants was recorded, by adding the N inputs from the stock solutions in the NS and subtracting the N remained at the drainage solution, at the end of the cropping period. Nitrogen uptake (mg plant^−1^ day^−1^) was calculated.

The water use efficiency [38] was determined and indicated as the irrigation water productivity (WP_I_) was calculated by the ratio between the marketable yield produced by a crop along the growing season and the irrigation water applied (IWU) in the same period, as described by Fernández et al. [39]:

(5) $$\text{Irrigation}\;\text{Water}\;\text{Productivity}=\frac{\text{Yield}\;\left(\text{kg}\;\text{per}\;\text{ha}\right)}{\text{IWU}\;\left(m^{3}\;\text{per}\;\text{ha}\right)}$$

The nitrogen use efficiency was indicated by the Agronomic Efficiency of N (AE_N_) and was calculated by the ratio of yield to N supply, as described by Ladha et al. [40]:

(6) $$\text{Agronomic}\;\text{Efficiency}\;\text{of}\;N=\frac{\text{Yield}\;\left(\text{kg}\;\text{per}\;\text{ha}\right)}{N\left(\text{kg}\;\text{per}\;\text{ha}\right)}$$

### 2.5 Total phenols, total flavonoids, antioxidant activity, ascorbic acid, and total soluble sugars

Polyphenols were extracted from six samples (two individual plants were pooled/sample) for each treatment. Methanolic extracts of the plant tissue (0.7 g) were stored at −20 ^ο^C until use for analysis of total phenolic and flavonoids content and total antioxidant activity by three assays. The 2,2-diphenyl-1-picrylhydrazyl (DPPH), the ferric reducing antioxidant power (FRAP) and the 2,2′-azino-bis(3-ethylbenzothiazoline-6-sulphonic acid (ABTS) methods were used.

Quantification of total phenols was made by using the methanolic extracts with the Folin–Ciocalteu method (using a microplate spectrophotometer Thermo Scientific, Multiskan GO), as described previously [41]. Briefly, plant extract was mixed with 125 μL of Folin reagent and 1.25 mL of 7 % Na_2_CO_3_, and after an incubation in the dark for 90 min, the absorbance was measured at 755 nm. Results were expressed in gallic acid equivalents (mg GA g^−1^ Fw). The content of total flavonoids was determined based on the aluminium chloride colorimetric method [42] as modified in Chrysargyris et al. [43]. The tested extracts were mixed with 5 % sodium nitrite (NaNO_2_) and 10 % AlCl_3_ solution. Then 0.5 mL of NaOH (1 M) solution was added. The absorbance was measured at 510 nm. The total flavonoid content was expressed in rutin equivalents (mg rutin g^−1^ FW).

Free radical-scavenging activity was determined as descripted previously [24]. In more details, DPPH radical scavenging activity of the plant methanolic extracts was measured at 517 nm. The reaction mixture contained plant extract and 0.3 mM DPPH solution. FRAP activity was assessed as described in Chrysargyris et al. [24]. The reaction mixture that contained 0.3 mM acetate buffer, pH 3.6), 10 mM TPTZ (Tripyridil-s-triazine), 40 mM FeCl_3_ 10H_2_O and plant extract was incubated at 37 °C for 4 min and the absorbance was measured at 593 nm. The ABTS assay was implemented according to the methodology described by Woidjylo et al., where plant extract was mixed with 7 mM ABTS solution and the absorbance was measured at 734 nm [44]. Results were expressed as Trolox ((±)-6-Hydroxy-2,5,7,8-tetramethylchromane-2-carboxylic acid) equivalents (mg trolox g^−1^ of fresh weight).

Ascorbic acid (AA) was determined by the 2,6-Dichloroindophenol titrimetric method [45]. Briefly, plant tissue (0.5 g) was extracted in 10 mL oxalic acid 4.0 % and was titrated by the dye solution until the color changed. Data were expressed as μg of AA per gram of fresh weight. Total soluble solids (TSS) concentration was determined from the juice obtained from plant tissue (n = 6) with a temperature-compensated digital refractometer (model Sper Scientific 300,017, Scottsdale, Arizona, USA) at 20 °C, and results were expressed in percentage (%).

### 2.6 Lipid peroxidation, hydrogen peroxide, and enzymes antioxidant activity

Hydrogen peroxide (H_2_O_2_) content was determined by using the method given by Loreto and Velikova [46] from six samples (two individual plants were pooled/sample) for each treatment. Frozen leaf tissue was grinded with 0.1 % tricloroacetic acid (TCA) and the produced extract was mixed with 0.5 mL of 10 mM potassium phosphate buffer pH 7.0 and 1 mL of 1 M KI. The H_2_O_2_ concentration was calculated using standards of 5–1000 μM of H_2_O_2_ and the calibration curve was plotted accordingly. The absorbance of samples and standards was measured at 390 nm and results were expressed as μmol H_2_O_2_ g^−1^ fresh weight.

Lipid peroxidation was assessed according to De Azevedo Neto et al. [47] and measured in terms of malondialdeyde content (MDA). The extract produced from the hydrogen peroxide content assay was used in this case as well. The extract was incubated with 0.5 % thiobarbituric acid (TBA) in 20 % TCA, at 95 °C for 25 min. The absorbance of the reaction mixture was then determined at 532 nm and corrected for non-specific absorbance at 600 nm. MDA amount was determined using the extinction coefficient of 155 mM cm^−1^. Results were expressed as nmol of MDA g^−1^ fresh weight.

For the antioxidant enzyme activities, fresh leaf tissue was homogenized using an ice-cold buffer containing 1 mM ethylenediaminetetraacetic acid (EDTA), 1 % (w/v) polyvinylpyrrolidone (PVPP), 1 mM phenylmethylsulfonyl fluoride (PMSF) and 0.05 % Triton X-100 in 50 mM potassium-phosphate buffer (pH 7.0). The activity of superoxide dismutase (SOD) (EC 1.15.1.1) and catalase (CAT) (EC 1.11.1.6) was assayed as described previously [48]. Briefly, SOD was assayed using a photochemical method; the reaction mixture containing 50 mM K-phosphate buffer (pH 7.5), 13 mM methionine, 75 μM nitro blue tetrazolium (NBT), 0.1 mM EDTA, 2 μΜ riboflavin and plant extract was exposed to a light source of two 15-W fluorescent lamps for 15 min. Then, the absorbance was determined at 560 nm, and the activity was expressed as units of SOD mg^−1^ of protein. For the catalase activity, the reaction mixture contained 50 mM K-phosphate buffer (pH 7.0), 10 mM H_2_O_2_ and plant extract. The decomposition of H_2_O_2_ was measured at 240 nm. The results were expressed as CAT units mg^−1^ of protein. Peroxidase activity (POD) (EC 1.11.1.6) was determined following the increase in absorbance at 430 nm as described previously [49], using 100 mM pyrogallol as substrate. Results were expressed as enzyme units per mg of protein. The protein content was determined by using bovine serum albumin as a standard.

### 2.7 Statistical methods

Analysis of variance (ANOVA) was performed on the data by IBM SPSS v.22, and results presented as treatment mean ± standard error (SE) of six biological measurements. Duncan's multiple range tests were performed when AVOVA rendered a significant treatment impact at *P* < 0.05. The correlation coefficients between Nr ratios with individual parameters tested as well as the correlation coefficients of nitrogen content in leaves and/or roots were also determined by Pearson's correlation test.

## 3 Results

### 3.1 Evolution of EC and pH of the drainage nutrient solution

**Fig. 1:**
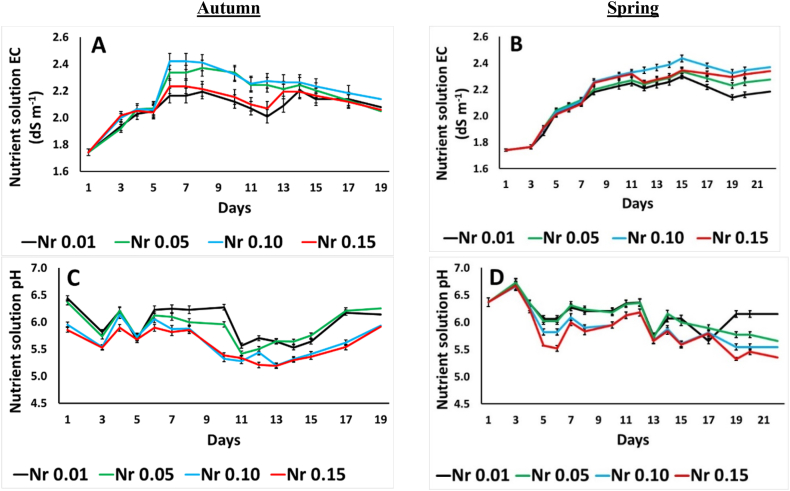
Effect of ammonium to total nitrogen ratio (NH_4_^+^/Total N= Nr 0.01–0.05-0.10–0.15) on the electrical conductivity (EC) and pH of the drainage nutrient solution from purslane plants grown hydroponically in NFT system in autumn (A,C) and spring (B,D). Error bars show SE (n = 3).

The evolution of pH and EC of the drainage nutrient solutions with the different Nr ratios applied to purslane plants during autumn and spring is presented in Fig. 1. The EC was fluctuated from 1.99 to 2.42 dS m^−1^ among the treatments during autumn and 1.86 to 2.38 dS m^−1^ among the treatments during spring (Fig. 1A and B). The pH of the NS drainage was significantly reduced during the most days of the cultivation period at the Nr study, especially for the high Nr ratios i.e., Nr 0.10 and Nr 0.15 (Fig. 1C and D).

### 3.2 Growth parameters

Data presented in Table 1 revealed that the plant growth related variables were differently influenced by the different Nr ratios provided to the plants thought the NS, during the two growing periods. In autumn, the higher upper plant and root biomass was revealed at low Nr (0.01), resulting in increased levels of dry weight (Table 1). However, during spring, plant growth related parameters were affected to a lesser degree by the applied Nr ratios. The ratio of Nr 0.15 decreased leaf fresh and dry weight, in comparison to Nr 0.05, and this was reflected to a lower plant biomass at the Nr 0.15 (Table 1).

**Table 1:** Effect of ammonium to total nitrogen ratio (NH_4_^+^/Total N= Nr 0.01–0.05-0.10–0.15) on plant height (cm), number of leaves, upper plant (leaves, stems) and roots fresh weight (FW; g) and dry weight (DW; g) of purslane plants grown hydroponically in NFT system under two seasons. Significant differences (*P* < 0.05) among Nr ratios are indicated by different letters.

	Plant height	Number of leaves	Leaves FW	Stems FW	Roots FW	Upper plant FW	Leaves DW	Stems DW	Roots DW	Upper plant DW
**Nr**					**Autumn**					
**Nr 0.01**	42.20a	303.60a	55.38a	89.32a	10.16a	155.42a	2.23a	4.01a	0.83a	6.24a
**Nr 0.05**	40.80 ab	236.80b	38.55b	55.40b	7.30b	97.74b	1.56b	2.51b	0.63b	4.07b
**Nr 0.10**	37.00b	201.60bc	30.00b	41.30b	5.74b	73.48b	1.18b	1.96b	0.48b	3.15b
**Nr 0.15**	38.41b	169.00c	34.92b	43.54b	6.38b	80.00b	1.31b	2.02b	0.48b	3.33b
**Nr**					**Spring**					
**Nr 0.01**	21.50	83.00	46.00a	27.76	35.00	70.53a	1.74a	0.78	1.75	2.54
**Nr 0.05**	20.33	96.00	40.06 ab	21.46	28.20	65.24 ab	1.48 ab	0.87	1.36	2.37
**Nr 0.10**	17.50	65.33	27.26 ab	14.60	18.15	44.84 ab	1.03 ab	0.61	0.85	1.66
**Nr 0.15**	18.83	65.66	23.36b	11.96	17.00	42.68b	0.88b	0.47	0.88	1.37

### 3.3 Physiological parameters

The different levels of ammonium to total N in the NS did not affect the physiology of purslane plants during autumn (Table 2). Therefore, leaf stomatal conductance was averaged at 52.53 mmol m^−2^ s ^−1^, leaf chlorophyll fluorescence (indicating maximal quantum yield of PSII photochemistry (F_v_/F_m_)) and SPAD units averaged at 0.764 and 32.27, respectively. Moreover, the content of chlorophyll *a*, chlorophyll *b*, and total chlorophylls were averaged at 33.56, 5.25, and 38.87 mg g ^−1^ fresh weight.

**Table 2:** Effect of ammonium to total nitrogen ratio (NH_4_^+^/Total N= Nr 0.01–0.05-0.10–0.15) on leaf stomatal conductance (mmol m^−2^ s ^−1^), chlorophyll content (*a*, *b*, total; mg g ^−1^ fresh weight), SPAD value, leaf chlorophyll fluorescence (F_v_/F_m_), electrolyte leakage (%) of purslane plants grown hydroponically in NFT system under two seasons. Significant differences (*P* < 0.05) among Nr ratios are indicated by different letters.

	Stomatal conductance	Chlorophyll *a*	Chlorophyll *b*	Total Chlorophylls	SPAD	Chlorophyll fluorescence	Electrolyte leakage
**Nr**				**Autumn**			
**Nr 0.01**	54.58	0.326	0.050	0.376	32.05	0.77	30.84
**Nr 0.05**	50.41	0.359	0.057	0.416	31.96	0.77	31.13
**Nr 0.10**	53.55	0.319	0.050	0.369	33.63	0.74	23.36
**Nr 0.15**	51.58	0.341	0.052	0.393	31.43	0.77	30.91
**Nr**				**Spring**			
**Nr 0.01**	51.00b	0.398a	0.104a	0.503a	32.20	0.73a	30.67
**Nr 0.05**	49.62b	0.227b	0.061b	0.288b	29.32	0.72a	29.78
**Nr 0.10**	68.10a	0.238b	0.060b	0.298b	28.80	0.68a	24.89
**Nr 0.15**	69.25a	0.348a	0.089a	0.438a	26.90	0.62b	30.23

Purslane grown during the spring period exhibited increased stomatal conductance at Nr ≥ 0.10. Chlorophylls content was higher at the low (Nr 0.05) and high (Nr 0.15) Nr ratios, while leaf chlorophyll fluorescence was decreased at the Nr 0.15 in comparison to the lower Nr ratios (Table 2).

Electrolyte leakage was similar during both seasons and under all the tried Nr ratios, averaged at 28.97 %.

The content of total phenols, total flavonoids, and antioxidant activity (as assayed by DPPH, FRAP, and ABTS) were decreased at Nr 0.15 in comparison lower Nr levels (i.e., Nr ≤0.10) during autumn (Table 3). At the highest Nr levels of 0.15, both ascorbic acid and TSS revealed the highest values, in comparison to lower Nr levels.

**Table 3:** Effect of ammonium to total nitrogen ratio (NH_4_^+^/Total N= Nr 0.01–0.05-0.10–0.15) on total phenols (mg GA g^−1^ FW), antioxidant activity [(2,2-diphenyl-1-picrylhydrazyl (DPPH), the ferric reducing antioxidant power (FRAP) and 2,2′-azino-bis(3-ethylbenzothiazoline-6-sulphonic acid (ABTS); mg Trolox g^−1^ FW)] and flavonoids (mg Rutin g^−1^ FW), ascorbic acid (μg g^−1^ FW) and total soluble sugars (TSS: %) of purslane plants grown hydroponically in NFT system under two seasons. Significant differences (*P* < 0.05) among Nr ratios are indicated by different letters.

	Total Phenols	DPPH	FRAP	ABTS	Flavonoids	AA	TSS
**Nr**			**Autumn**				
**Nr 0.01**	0.297a	0.221a	0.251a	0.097a	0.195a	3.582c	2.000b
**Nr 0.05**	0.288a	0.213a	0.240a	0.085a	0.193a	7.608 ab	2.033b
**Nr 0.10**	0.289a	0.232a	0.247a	0.088a	0.154a	5.881bc	1.766c
**Nr 0.15**	0.226b	0.172b	0.168b	0.056b	0.135b	8.932a	2.766a
**Nr**			**Spring**				
**Nr 0.01**	0.103	0.206	0.645a	0.298a	0.332a	2.133c	3.930
**Nr 0.05**	0.103	0.267	0.649a	0.286 ab	0.315 ab	2.200bc	4.686
**Nr 0.10**	0.079	0.228	0.453b	0.237c	0.092c	2.266b	6.085
**Nr 0.15**	0.087	0.190	0.565 ab	0.246bc	0.260b	2.467a	5.201

During spring, purslane antioxidant activity (as assayed by FRAP and ABTS) as well as total flavonoids content were decreased at the high Nr levels. However, AA content appeared to increase at the highest tested Nr ratio of 0.15 (Table 3). Total phenols content remained to similar levels among the different Nr ratios.

Considering the effect of the Nr ratios during autumn, the MDA content appeared increased at the low Nr (0.01) and high Nr (0.15), in comparison to the Nr 0.05 (Table 4). However, the H_2_O_2_ production was similar to all the examined Nr levels, averaged at 0.183 μmol g^−1^ fresh weight. SOD and CAT activities were increased at Nr 0.15 while POD activity was increased at Nr 0.10.

**Table 4:** Effect of ammonium to total nitrogen ratio (NH_4_^+^/Total N= Nr 0.01–0.05-0.10–0.15) on hydrogen peroxide (H_2_O_2_; μmol g^−1^), lipid peroxidation-malondialdeyde content (MDA; nmol g^−1^) and antioxidant enzymes activity of superoxide dismutase (SOD; units mg^−1^ protein), catalase (CAT; units mg^−1^ protein), and peroxidase (POD; units mg^−1^ protein) of purslane plants grown hydroponically in NFT system under two seasons. Significant differences (*P* < 0.05) among Nr ratios are indicated by different letters.

	H_2_O_2_	MDA	SOD	CAT	POD
**Nr**			**Autumn**		
**Nr 0.01**	0.166	6.52a	0.49b	7.63b	0.93b
**Nr 0.05**	0.192	4.82b	0.42c	7.30b	0.78b
**Nr 0.10**	0.161	5.41 ab	0.44bc	8.38b	1.11a
**Nr 0.15**	0.212	6.14a	0.56a	11.18a	0.88b
**Nr**			**Spring**		
**Nr 0.01**	0.312	11.52a	1.16c	24.99b	1.99b
**Nr 0.05**	0.327	10.01 ab	1.13c	24.52b	1.97b
**Nr 0.10**	0.306	8.75b	1.30b	29.78a	1.60c
**Nr 0.15**	0.328	10.07 ab	1.48a	30.46a	2.51a

During spring, MDA increased at the lowest Nr ratio of 0.01, while the activities of SOD, CAT and POD increased at the high Nr (≥0.10) (Table 4). Similar to autumn, the H_2_O_2_ production was unaffected by the Nr levels during spring.

### 3.4 Leaf, stem and root nutrient content

**Fig. 2:**
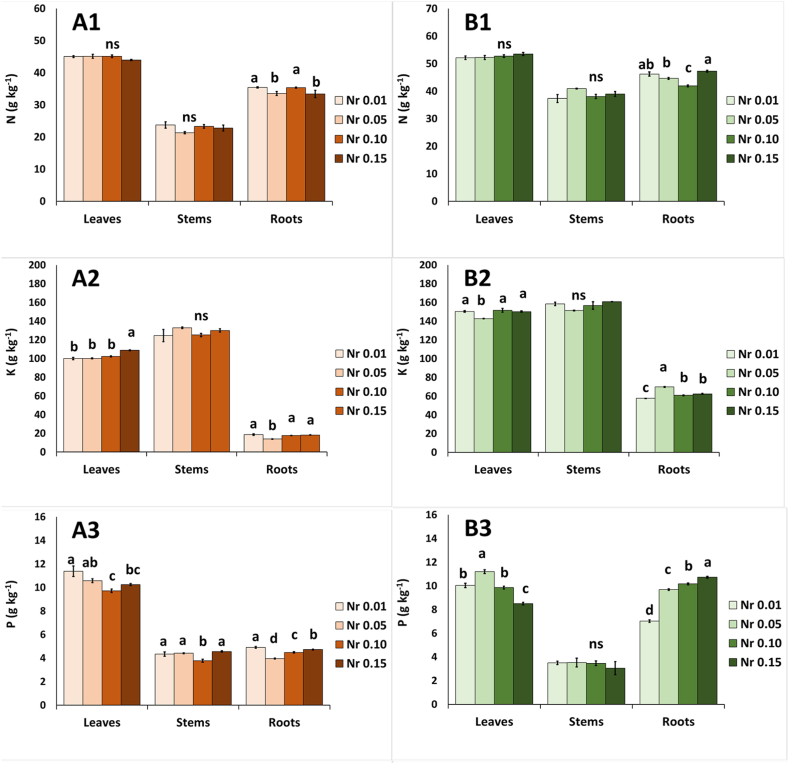

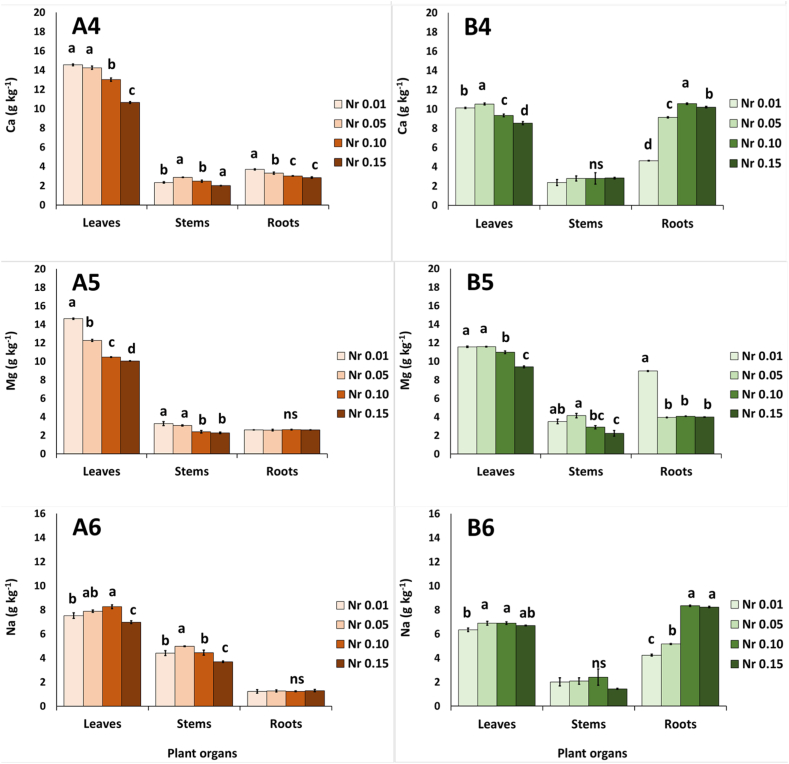
Effect of ammonium to total nitrogen ratio (NH_4_^+^/Total N= Nr 0.01–0.05-0.10–0.15) in the nutrient solution on the content of macronutrients in leaves, stems and roots of purslane plants grown hydroponically in NFT system under two seasons (autumn: A and spring: B). (A1,B1) nitrogen-N, (A2,B2) potassium-K, (A3,B3) phosphorus-P, (A4,B4) calcium-Ca, (A5,B5), magnesium-Mg, and (A6,B6) sodium-Na. Significant differences (*P* < 0.05) among Nr ratios in each plant organs, are indicated by different letters; ns indicates non-significant. Error bars show SE (n = 4).

During autumn, the Nr affected the content of macronutrients in leaves, stems and roots (Fig. 2). Nitrogen was accumulated in roots at Nr 0.01 and Nr 0.10, but no differences were found in the upper part of the plant, leaves and stems (Fig. 2A1). In leaves, potassium was significantly higher at Nr 0.15 in comparison to lower Nr levels (≤0.10); in roots, potassium content had the lowest value at Nr 0.05 in comparison to lower or higher Nr ratios (Fig. 2A2). Leaf phosphorus content was decreased in Nr ≥ 0.10 in comparison to lower ratio of Nr 0.01 (Fig. 2A3). In stems, phosphorus revealed the lowest accumulation at Nr 0.10 (Fig. 2A3), while this value was decreased at the increased levels of Nr in roots (Fig. 2A3). Calcium content appeared to decrease in leaves and roots with the increase of Nr ratio (Fig. 2). Magnesium level was decreased in leaves and stems when the Nr ratio was increased (Fig. 2A5), while no differences were observed on the magnesium content of the roots. Sodium was highly accumulated in roots as the Nr levels were increased (Fig. 2A6), or in leaves, at the middle levels of Nr (i.e., Nr 0.05–0.10).

During spring season, the content of macronutrients in leaves, stems and roots was also affected by the applied ratios of Nr (Fig. 2). Nitrogen was accumulated in roots at Nr 0.15 in comparison to Nr 0.05 and Nr 0.10, but no differences were found in the upper plant tissue, leaves and stems (Fig. 2B1). Potassium content was significantly decreased at Nr 0.05 in leaves but was increased in roots at the same Nr levels of 0.05 (Fig. 2B2). Leaf phosphorus content was increased at Nr 0.05, while it was accumulated in roots as the Nr ratio in the NS was increased (Fig. 2B3). Calcium content appeared to increase at Nr 0.05 in the leaves and at the Nr 0.10 in the roots (Fig. 2B4). Magnesium content was decreased in all plant parts as the ratio of Nr was increased (Fig. 2B5). Sodium accumulated more in leaves and stems at Nr 0.05 in comparison to lower or higher Nr levels (Fig. 2B6).

### 3.5 Nutrient uptake concentration and use efficiency

During autumn, nutrient uptake decreased as the Nr increased (Table 5). The highest water uptake was found at Nr 0.15 and the lower at Nr 0.05. The WUE was significantly higher at Nr 0.01 in comparison to Nr ≥ 0.10. The NUE revealed a decreased trend as the Nr increased, however these changes did not differ significantly.

**Table 5:** Influence of ammonium to total nitrogen ratio (NH_4_^+^/Total N= Nr 0.01–0.05-0.10–0.15) on nutrient uptake (mL plant^−1^), water uptake (L plant^−1^), nitrogen use efficiency (NUE; g DW mL^−1^ NS), water use efficiency (WUE; g DW L^−1^ H_2_O) of purslane plants grown hydroponically in NFT system under two seasons. Significant differences (*P* < 0.05) among Nr ratios are indicated by different letters.

	Nutrient uptake	Water uptake	NUE	WUE
**Nr**		**Autumn**		
**Nr 0.01**	36.97a	8.12 ab	0.16	0.73a
**Nr 0.05**	31.48bc	8.04b	0.13	0.51 ab
**Nr 0.10**	29.28c	8.24 ab	0.11	0.39b
**Nr 0.15**	33.68b	8.88a	0.10	0.38b
**Nr**		**Spring**		
**Nr 0.01**	16.92a	6.58a	0.15a	0.38b
**Nr 0.05**	14.66b	5.20b	0.16a	0.45a
**Nr 0.10**	14.64b	5.61b	0.11b	0.29c
**Nr 0.15**	14.94b	5.33b	0.09b	0.25d

During spring, both nutrient and water uptake decreased at Nr ≥ 0.05 (Table 5). The calculated NUE was higher at Nr 0.01 and Nr 0.05 but appeared decreased at Nr ≥ 0.10. The WUE was significantly higher at Nr 0.05, followed by Nr 0.01, and then by Nr ≥ 0.10.

Ammonium to total N ratio affected the transfer of N, as the nitrogen accumulation rate decreased as the Nr ratio was increased in the NS, during autumn (Table 6). All bioaccumulation coefficients for leaves, stems, and roots were significantly decreased at the Nr ≥ 0.05 in the NS during autumn, with more prompt decrease to be evident at the higher Nr ratios. The translocation factor of N from the roots to the stems received higher value at Nr 0.15 in comparison to the Nr 0.05, however, N translocation factor was not differed in the case of leaves. The applied Nr ratios affected the calculated TI for the different plant growth-related parameters. The TI of the number of produced leaves and the total plant biomass decreased at Nr ≥ 0.05, and therefore these of the fresh and dry weight of leaves and stems. Indeed, the TI for plant height was decreased at the Nr 0.10 in comparison to Nr 0.01.

**Table 6:** Influence of ammonium to total nitrogen ratio (NH_4_^+^/Total N= Nr 0.01–0.05-0.10–0.15) on the nitrogen accumulation rate-AR (g kg^−1^ Dw day^−1^), bioaccumulation coefficient-BAC, translocation factor-TF and tolerance indices-TI (%) of purslane plants grown hydroponically in NFT system under two seasons. Significant differences (*P* < 0.05) among Nr ratios are indicated by different letters.

	Accumulation rate	Bioaccumulation coefficient			Translocation factor		Tolerance indices						
		Leaves	Stems	Roots	Leaves	Stems	Total Biomass	Plant height	Leaf No	Fresh leaves	Fresh Stems	Dry leaves	Dry Stems
**Nr**					**Autumn**								
**Nr 0.01**	48.40a	222.88a	117.48a	175.47a	1.27	0.67 ab	100.00a	100.00a	100.00a	100.00a	100.00a	100.00a	100.00a
**Nr 0.05**	20.53b	214.42b	101.40b	159.37b	1.34	0.63b	62.88b	96.68 ab	77.99b	69.62b	62.02b	69.97b	62.58b
**Nr 0.10**	13.06c	202.98c	104.80b	159.26b	1.27	0.66 ab	47.27b	87.68b	66.40bce	54.17bc	46.23b	52.99c	49.05b
**Nr 0.15**	12.26c	186.91d	96.87b	142.05c	1.31	0.71a	51.47b	93.36 ab	55.66c	63.05c	48.74b	58.71bc	50.40b
**Nr**					**Spring**								
**Nr 0.01**	28.11a	257.96a	184.86 ab	228.76a	1.12c	0.81b	100.00a	100.00a	100.00a	100.00a	100.00a	100.00a	100.00a
**Nr 0.05**	19.60b	237.22c	171.40bc	188.64d	1.25a	0.91a	92.49a	48.18b	31.62b	72.34b	24.03b	66.13b	21.72b
**Nr 0.10**	12.89c	248.19b	194.53a	212.08b	1.17b	0.92a	53.34b	41.47b	21.52c	49.23c	16.34bc	46.32c	15.23bc
**Nr 0.15**	8.58d	227.36d	165.64c	200.64c	1.13c	0.82b	60.49b	44.62b	21.63c	42.19c	13.39c	39.62c	11.73c

During spring growing period, nitrogen accumulation rates were decreased as the Nr in the NS was increased (Table 6). Leaf and root bioaccumulation coefficients were significantly decreased with the increase of Nr, while stem bioaccumulation coefficient was increased at the Nr 0.10. The translocation factor of N from the roots to the leaves and from the roots to the stems received the higher value at Nr 0.05 and Nr 0.05–0.10, respectively, in comparison to the lower or higher Nr levels. The Nr ratio affected TI for the different plant growth-related parameters, as TI was decreased at Nr ≥ 0.10 for the plant biomass and at Nr ≥ 0.05 for the plant height and number of produced leaves, resulting in decreased values for the fresh and dry weight of leaves and stems as well.

### 3.6 Correlation of Nr ratios to individual parameters

Linear correlation coefficients were calculated and reported in detail on Tables S2–S3, to describe the contribution of the Nr levels in the NS for each individual parameter. The correlation coefficient (r) and p-values are also given. Considering the impacts of the Nr in the NS during the growing period of autumn, a positive correlation of Nr with ascorbic acid content, TSS, CAT activity and leaf potassium content was observed. On the other hand, it was revealed a negative correlation with number of leaves, fresh and dry weight of plant (leaves, stems, roots), total phenols, total flavonoids, antioxidant capacity (assayed by FRAP, ABTS), leaf phosphorus, magnesium, and calcium content, stem sodium and magnesium content, and root calcium content (Table S2). The nitrogen accumulation in leaves was positively correlated to the stem's dry matter and negatively correlated to TSS, CAT and SOD enzyme activities.

As for the spring period, a positive correlation of Nr with SOD and POD activities, ascorbic acid content, root phosphorus, sodium and calcium content were noticed, while features as leaf chlorophyll fluorescence, leaf phosphorus, magnesium and calcium content, stem magnesium content and root magnesium content were negatively correlated (Table S3). The nitrogen accumulation in leaves was negatively correlated with leaf dry weight.

## 4 Discussion

As the form of N taken up by the plants affects efficiency and metabolism of N in plants, in this study we aimed to examine the response of purslane plants to different NH_4_/NO_3_ ratios. Purslane plants were cultivated under different ammonium to total nitrogen ratios, during two successive growing periods (autumn and spring). Purslane can perform better at temperatures ranging from 18 to 32 °C, even though it can tolerate a temperature range between 7 and 36 °C [50]. There are several reports indicating that the management of the NS composition in soilless cultivation systems can be considered an important strategy for improving the nutritional quality of the produced vegetables [43,48,51,52]. Therefore, as the nitrogen source for plants in a hydroponic nutrient solution is ammonium and/or nitrates, the relevant Nr ratios of 0.01, 0.05, 0.10 and 0.15, were employed to acquire insights in the *P. oleracea* performance in hydroponics. The effects of NH_4_ and NO_3_ supply on plant growth have been the subject of several research studies, and interestingly, the obtained results appear to be variable and largely dependent on the species and the cultivation techniques employed. When it comes to plant growth and the synthesis/accumulation of chemical compounds, it is always preferred to combine nitrate and ammonium nutrition than to use solitary sources of NO_3_ or NH_4_, for most plants, given that the NS is kept at a pH close to neutral, even slightly acidic (i.e. pH 5.8) [11,12]. In order to achieve this, in the present study a mixed nitrate and ammonium fertigation was employed, while keeping the total N levels constant, at 200 mg L^−1^, using a hydroponic cropping system. These systems are in place for the precision management of the mineral fertigation, and have been shown to create quality standards for the crops [53]. The findings of the present study showed that plant growth, physiology/biochemistry, mineral accumulation, and the efficiency of nitrogen and water consumption were all influenced by the different N sources (different Nr ratios) in the NS.

Generally, it is believed that a plant's inability to undertake photosynthesis, lack of carbon skeletons, and increased ammonium accumulation are reasons that cause losses in terms of plant growth and production, driven on by low amounts of N-nitrate or high levels of N-ammonium [9]. However, ammonium at excessive levels can be harmful to plants, reduce the production of organic acids, and impair osmotic regulation [54]. The importance of this parameter was examined and proved in the present study. Results revealed that plant growth related parameters (plant height, leaves, and plant fresh biomass) were decreased while the Nr ratio was increased; these effects were more profound during autumn period than early spring. Similarly, Song et al. [55] reported that plant height and biomass were declined under high Nr ratios in comparison to lower Nr levels, when flowering Chinese cabbage was cultivated under different Nr in hydroponics. In contrast, Zhu et al. [12] reported increased leaf area, photosynthetic rates and biomass in *Prunella vulgaris* (L.) grown at Nr 0.2, while Savvas et al. [10] reported changes on fresh biomass of lettuce plants when subjected to Nr 0.1 or Nr 0.2, in a closed hydroponic system. Although greater Nr levels (i.e. Nr 0.5) reduced yield and resulted in toxicity symptoms, the Nr applied in the current study are even less than the maximum acceptable levels of 0.23, that were tested in the NS for iceberg lettuce in a floating-hydroponic system [9]. When no ammonium was provided to medicinal cannabis, it has been reported a higher crop production, increased transpiration, photosynthesis rates, stomatal conductance, and chlorophyll content, and increased secondary metabolism under nitrate nutrition [56]. In the case of purslane, the highest purslane upper biomass was found in plants exposed to only nitrates (at Nr 0.0) [57], and this is in line with the present finding, when greater biomass was revealed at the lowest examined Nr of 0.01. However, ammonium presence in the NS is preventing the nitrate build-up inside the plant tissue, maintains the pH of the NS and occasionally improves fresh produce quality [14]. Purslane's low production at higher Nr ratios is a result of the plant's slow growth and development of its leaves, which is additionally supported by the lower total number of leaves. This may be due to the ammonium toxicity to plants or to rhizosphere acidification, where under high ammonium concentration conditions, plant root releases H^+^ during absorption of ammonium in order to maintain the cation/anion balance [50]. This is well documented, as a response to stress conditions, as there are researches reporting that salinity and ammonium can reduce biomass production and oxalic acid content in *P. oleracea* [58].

Purslane grown in Deep Flow Technique (DFT) system during spring had greater total fresh and dry plant weights in comparison to plants grown during autumn [59]. These findings are in contrast with the present study, as greater plant biomass was observed during autumn period than spring. However, these differences can be attributed to the different hydroponic system (NFT vs DFT), the different composition of the nutrient solution, as well as the cultivation period dates (autumn for October-early November in Cyprus and November–December in Turkey), while the spring period was more or less similar (March–April), and the different conditions during the trials. It is important to note that while the optimal Nr levels in the present investigation are applicable for the observed spectrum of root system temperatures, which was ranged from 19.2 to 24.7 °C, as recorded by the pH/EC devise on a daily basis, plant sensitivity to ammonium toxicity raises along with a rise in root zone temperature [60].

The level of photosynthetic activity in leaves is influenced by changes in the amount of chlorophylls, the composition of the chloroplasts, and the stomatal conductance [61]. The ratio of F_v_/F_m_ is an important feature for plants, since it demonstrates the appropriate function of the light reaction and is employed to evaluate how and if any stress condition is affecting the tested plants. In many species, a recorded F_v_/F_m_ value inside the range of 0.79–0.84 is typical [62]. Changes in the photosynthetic performance were only observed during the spring period. Increased stomatal conductance was measured on plants cultivated at high Nr levels, with decreased F_v_/F_m_ values of the maximal quantum yield of PSII. Chlorophyll content was increased at low (Nr 0.01) and high (Nr 0.15) ratios of ammonium to total nitrogen, but remained low at Nr 0.05–0.10. Another study on medicinal plant *Dracocephalum moldavica* L [63]. revealed also the same trend; higher NO_3_ and NH_4_ levels both increased photosynthetic pigments and this may be due to unrevealed matters in uptake of both forms of N. This may results from the fact that high NH_4_ content promotes nitrogen uptake and translocation of N to the leaves and inflorescence, and thus it has a direct effect on N metabolism. In that way, it interferes to the photosynthetic activity resulting in a decrease in carbohydrate production, unstable root homeostasis, oxidative stress, and growth reduction, as it was mentioned previously [56]. However, purslane exposed to higher Nr levels of 0.25 up to 0.75 revealed decreased chlorophyll content and curly leaves [57]. Such macroscopic symptoms were absent for the present study due to the lower range of Nr levels used.

Edible plant material with a high phenolic content can prevent tissue oxidation by scavenging free radicals and inhibiting lipid peroxidation, improving the nutritional value of the edible part, and removing potential issues caused by an excessive intake of synthetic additives, which is why phenolic-containing plant parts and their products are of great interest [64]. As a result, tissues rich in phenolic compounds have the ability to protect against the damage caused by oxidative stress brought on by free radicals. Saadatian et al. [65] reported that the Nr 0.11 resulted in increased phenolic content and antioxidants levels (DPPH) in comparison to the higher tested ratios (Nr 0.20 and Nr 0.27) in hydroponically (NFT) grown basil, being in agreement with the present study, as purslane revealed increased phenolic content at even lower Nr levels (Nr 0.01). According to Naseri et al. [63] decreasing of phenolics content in plant tissues (leaves and roots) due to the supply of NH_4_ as sole N source reported in several plant species (pea, corn, etc.). In this case, ammonium may by altering intracellular acidity affects the biosynthesis of metabolites, including the phenylpropanoid pathway, that produces a series of secondary metabolites, directly correlated to the antioxidant activity. During autumn, purslane total phenolic content was almost three times higher than the relevant values during spring. According to Lim and Quah's research, there was a significant change in the total phenolic compound accumulation impacting the antioxidant capacity of purslane, which was directly related to the season of the year, as they are influenced by the environmental factors of the collection period, as temperature and sunlight [66].

One of the most important secondary medicinal metabolites, flavonoids have a high antioxidant capacity, making them well known for curing diseases, cardiovascular disorders, cognitive issues, and preventing the damage that free radicals cause to cell membranes. Under biotic and abiotic stresses, as well as nutritional deficiency conditions, these components are normally synthetized via photosynthesis to protect the plant from potential injury from free radicals [67,68]. According to Petropoulos et al. [68], purslane is one of the plants that contains flavonoids with a high antioxidant potential, as apigenin, kaempferol, luteolin, quercetin, myricetin, genistein, and portulacanones A to D. Moreover, Zhu et al. [12] reported increased flavonoids and rosmarinic acid in *Prunella vulgaris* (L.) grown at Nr 0.2, while Naseri et al. [63] reported increased flavonoid content at the lower ratios. Purslane's flavonoids content was increased at lower Nr levels (Nr ≤ 0.10 for autumn and Nr ≤ 0.05 for spring); and as it was mentioned above, increased ammonium content changes the intracellular acidity. That affects the phenylpropanoid pathway, which serves as a rich source of metabolites in plants, and as a starting point for the production of important compounds, such as the flavonoids [69].

Malondialdehyde, a naturally occurring byproduct of lipid peroxidation, has historically been employed as a marker of the degree of cell damage brought on by stress [70]. Plant activates detoxification mechanisms towards the reactive oxygen species (ROS) production by scavenging the free radicals with the induction of the activity of antioxidant enzymes as SOD, CAT, ascorbate peroxidase (APX), and glutathione reductase (GR) [71] or induction of non-enzymatic antioxidant mechanisms such as production of polyphenols, ascorbic acid, proline, etc. In the present study the increased MDA content was evidenced both in low (Nr 0.01) and high (Nr 0.15) ammonium ratio in the NS, indicating a situation of oxidative stress occurring inside the plants. This resulted in the activation of both non-enzymatic (ascorbic acid) and enzymatic (SOD, CAT and POD) antioxidant responses as a protective mechanism by the plant, to detoxify the stress conditions, as in other reports on the effect of high ammonium levels on *Salvia* species [72]. The activity of all antioxidant enzymes of purslane appeared to increase at the higher ratio of Nr. In that sense, the increased NH_4_ levels produce toxicity-induced osmotic changes that can boost the excessive accumulation of O_2_^−^ and H_2_O_2_, accordingly, resulting in higher lipid peroxidation. For the regulation of the increased hydrogen peroxide content SOD appears the most important and in the first line of defense, as a direct scavenger aided by CAT and POD [73]. Both TSS and AA were increased at the high Nr levels, further proving the physiological response to the ammonium stress. Indeed, TSS, proline, AA and total phenolics can be increased for protection against hyperosmotic stress [74], indicating the increased stress condition for purslane that occurred under the high Nr levels in the NS.

The increased Nr ratio affected the content of macronutrients in leaves, stems and roots. Mineral levels in the present study were within the levels reported in previous studies [75]. In the present study, the total N levels in leaf (averaged in 4.5–5.2 %) and in stem (averaged in 2.3–4.0 %), were higher than the adequate N levels (i.e. 1.5 %) that may be required by plants [76]. Total nitrogen content in leaves was not affected by the Nr levels in both growing seasons. However, in previous studies on *Prunella vulgaris* [12] and in Chinese kale (*Brassica alboglabra* L. H. Bailey) grown in high Nr [54], nitrogen content was increased at high Nr levels. This may not be the case for purslane, as the level of ammonium in the ratios used did not cause that trend. In the present study, potassium and sodium were accumulated more but phosphorus was less accumulated in the leaves of purslane grown at the high Nr levels in the NS. This may be due to the fact that high supply of ammonium affects the balance of N, K and P in plant tissue, resulting in a decrease in content in minerals as the Mg, which is a cation and can antagonise the other cations efficiency i.e., NH_4_, Ca, K [63]. Contrasting findings were observed in Chinese kale treated with high Nr ratio [54]. Despite the well documented competition between potassium and NH_4_^+^ cations for uptake by the roots [56], in the present work this is not evidenced for potassium. The accumulation of K noted in cases of high Nr ration, might be used by the plant as an alleviate mechanism to NH_4_^+^ toxicity [77]. A competition among NH_4_^+^ and calcium and magnesium cations could be noted, as both calcium and magnesium were less accumulated in leaves at the high Nr levels (meaning higher levels of NH_4_^+^). The pH of the drainage NS had a decreased tendency as the Nr was increased, being always within the acceptable pH limits and varied from 5.5 to 6.6, due to daily management of pH. Decreased pH at increased Nr levels in the NS has been previously reported for studies on lettuce [10], tomato [14] and *Cichorium spinosum* [16].

According to a series of studies, purslane leaves can be a great source of medicinal compounds and antioxidants, while the concentration of these compounds is increasing together with the nitrogen content in the leaves [78,79]. Moreover, purslane contains many dietary minerals such as potassium, magnesium, calcium, phosphorus, iron etc. Srivastava et al. found that potassium is the most abundant nutrient present in the plant (494 mg per 100 g fresh weight), and additionally projected the importance of it for a proper heart function [50]. Generally, in the present study, the tested purslane exhibited high nutritive value, as potassium was averaged in 403 and 506 mg per 100 g fresh weight, in leaves and stems, during autumn and in 555 and 588 mg per 100 g fresh weight, in leaves and stems, during spring, respectively. Additionally, there are reported cases where under ammonium nutrition (Nr 0.25–0.75), some amino acids, including glutamate, glycine, ornithine, phenylalanine, proline, serine, and tyramine, showed increased accumulation [57].

Indeed, Nr levels not only affected the growth and the secondary metabolism of the plant but also the use efficiency of the minerals into the crop production. Therefore, both NUE and WUE appeared high at Nr 0.01 during autumn and Nr ≤0.05 during spring. Given the current critical conditions for conserving water and fertilizers, because of several environmental and health-related issues, Nr of 0.05 can meet more efficiently with this demand, as that also results in high NUE during spring as well. The losses of nitrogen in a closed hydroponic system, as the one used in the current study, may be caused by variations in the nutrient solution's composition or by the plants' actual N metabolism [54]. Despite being able to narrow in more on that topic with the current study, the agronomic effectiveness of N in a closed hydroponic system is, however, an untapped yet poorly understood area for research. However, a detailed recording of the NH_4_^+^ and NO_3_^−^ levels variation during plant growth, could provide more sufficient information regarding the nitrogen metabolism pathways and formation of possible volatile forms of gaseous nitrides, including NH_3_, N_2_O, and NO [54].

Nitrogen accumulation rate was decreasing as the Nr ratio was increased in the NS, while nitrogen was less bioaccumulated in leaves, stems and roots at the increased Nr, in comparison to lower Nr ratios, during autumn. Interestingly, nitrogen bioaccumulation was higher in stems at the relevant high levels of Nr 0.10. The decreased translocation factor of nitrogen from the roots to the leaves, as the Nr ratio was increased, resulted in decreased plant biomass and generated oxidative stress as the MDA production was increased in high Nr ratios. The TF and BAC indices are useful factors in determining how effective a plant might be in phytoremediation and element accumulation on specific organs. Both parameters in hyperaccumulator species are greater than 1; as indicated for purslane leaves, but not for purslane stems, in the present study. The TF was high in the middle or high Nr levels, indicating the promotion of N to move from the roots to the leaves, but not from the roots to the stems.

The accumulation of nitrogen in leaves and roots under different Nr ratios revealed changes on plant growth and on accumulation of nutrients inside the plant organs. Nitrate metabolism is less efficient energetically than ammonium metabolism because nitrate must be firstly reduced to ammonium in order to be digested, and nitrate absorption and reduction are energy-intensive processes [56]. Therefore, oversupply of N or unbalanced ammonium to nitrate sources may have a strong impact on plant metabolism and function, affecting root ion balance and inhibiting or promoting the uptake and accumulation of other nutrients.

## 5 Conclusions

Our results have demonstrated that purslane, as an under-exploited vegetable but with important nutritive and pharmaceutical value, can cope with soilless culture and may be affected positively or negatively by the Nr application. Nitrogen is considered as a main nutrient, necessary for plant growth and development. However, the source of nitrogen (ammonium versus nitrate) is an even more important factor to consider, rather than the nitrogen concentration in the nutrient solution, for the hydroponically grown crops. In this study, the ammonium to total nitrogen ratio, as indicated by the Nr value, whereas plants revealed the less stressed condition and the higher mineral accumulation in leaves, was the Nr 0.05. For successful plant production, that is both safe for human consumption and friendly to the environment, it is essential to balance the increased growth in the upper plant part (biomass) with the production of high nutritive value products (i.e. rich in antioxidants, minerals), to sustain water and to control nitrogen fertilizers. Towards this direction, what is required is a careful nutrient solution management.

## Data availability

Data will be made available on request.

## CRediT authorship contribution statement

**Antonios Chrysargyris:** Conceptualization, Data curation, Investigation, Methodology, Supervision, Writing – original draft, Writing – review & editing. **Efraimia Hajisolomou:** Data curation, Formal analysis, Investigation. **Panayiota Xylia:** Data curation, Investigation, Methodology, Software. **Nikolaos Tzortzakis:** Conceptualization, Data curation, Funding acquisition, Project administration, Resources, Software, Supervision, Validation, Visualization, Writing – original draft, Writing – review & editing.

## Declaration of competing interest

The authors declare that they have no known competing financial interests or personal relationships that could have appeared to influence the work reported in this paper.
