# Supplementary material for: Ammonium to total nitrogen ratio affects the purslane (Portulaca oleracea L.) growth, nutritional, and antioxidant status
Source: Heliyon. 2023 Nov 2;9(11):e21644. doi: 10.1016/j.heliyon.2023.e21644 (PMC10661198; doi:10.1016/j.heliyon.2023.e21644)
Supplement: Multimedia component 1 [file mmc1.docx]

**Table S1**. Electrical conductivity (EC) and nutrient concentrations in the nutrient solution (NS) supplied to purslane plants grown in a closed hydroponic system. The concentration of Cl^-^ and Na^+^ in the primary water was 1.49 mM and 1.91 mM, respectively, and the pH of the nutrient solution was set at 5.8 using H_2_SO_4_ (5% v/v).

| Parameters | Nr 0.01 | Nr 0.05 | Nr 0.10 | Nr 0.15 |
| --- | --- | --- | --- | --- |
| EC dS m^-1^ | 2.32 | 2.32 | 2.32 | 2.34 |
| K^+^ mmol L^-1^ | 8.95 | 8.95 | 8.95 | 8.95 |
| Ca^2+^ mmol L^-1^ | 3.74 | 3.74 | 3.74 | 3.74 |
| Mg^2+^ mmol L^-1^ | 2.88 | 2.88 | 2.88 | 2.88 |
| NH_4_^+^ mmol L^-1^ | 0.14 | 0.71 | 1.43 | 2.14 |
| NO_3_^-^ mmol L^-1^ | 14.14 | 13.57 | 12.86 | 12.14 |
| SO_4_^2-^ mmol L^-1^ | 2.65 | 3.22 | 3.94 | 4.65 |
| H_2_PO_4_^-^ mmol L^-1^ | 2.26 | 2.26 | 2.26 | 2.26 |
| Cl^-^ mmol L^-1^ | 1.49 | 1.49 | 1.49 | 1.49 |
| Fe μmol L^-1^ | 71.56 | 71.56 | 71.56 | 71.56 |
| Mn μmol L^-1^ | 18.21 | 18.21 | 18.21 | 18.21 |
| Zn μmol L^-1^ | 1.53 | 1.53 | 1.53 | 1.53 |
| Cu μmol L^-1^ | 4.72 | 4.72 | 4.72 | 4.72 |
| B μmol L^-1^ | 18.52 | 18.52 | 18.52 | 18.52 |
| Mo μmol L^-1^ | 0.52 | 0.52 | 0.52 | 0.52 |

The modified NS of increasing ammonium to total nitrogen ration (NH_4_^+^/Total N= Nr 0.01-0.05-0.10-0.15) was applied after 1 week of plants grown in NFT. The partial increase of NH_4_^+^ was through the adding ammonium nitrate, the substitution of nitrate (NO_3_ ^-^) was observed by using sulphate (SO_4_^2-^) in the NS, and the total N level was constant of the 200 mg L^-1^.

The fertilizers used were: calcium nitrate, potassium nitrate, ammonium nitrate, magnesium sulphate, magnesium nitrate, potassium sulphate, phosphoric acid, nitric acid, sulphuric acid, iron-chelate, manganese sulphate, zinc sulphate, copper sulphate, boric acid and ammonium heptamolybdate.

**Table S2.** Correlations coefficients and (*p*-values) between the Nr levels in the NS and the nitrogen accumulation in plant organs during Autumn with the tested parameters.

**Table S3.** Correlations coefficients and (*p*-values) between the Nr levels in the NS and the nitrogen accumulation in plant tissue during Spring with the tested parameters.
